# CNF1 Enhances Brain Energy Content and Counteracts Spontaneous Epileptiform Phenomena in Aged DBA/2J Mice

**DOI:** 10.1371/journal.pone.0140495

**Published:** 2015-10-12

**Authors:** Sara Travaglione, Giulia Ballan, Andrea Fortuna, Alberto Ferri, Marco Guidotti, Gabriele Campana, Carla Fiorentini, Stefano Loizzo

**Affiliations:** 1 Department of Therapeutic Research and Medicines Evaluation, Istituto Superiore di Sanità, Viale Regina Elena 299, 00161, Roma, Italy; 2 Institute of Cellular Biology and Neurobiology, CNR, Via del Fosso di Fiorano 64/65, 00143, Roma, Italy; 3 Department of Veterinary Public Health and Food Safety, Viale Regina Elena 299, 00161, Roma, Italy; 4 Department of Pharmacy and Biotechnology, University of Bologna, Via Irnerio 48, 40126, Bologna, Italy; University of Modena and Reggio Emilia, ITALY

## Abstract

Epilepsy, one of the most common conditions affecting the brain, is characterized by neuroplasticity and brain cell energy defects. In this work, we demonstrate the ability of the *Escherichia coli* protein toxin cytotoxic necrotizing factor 1 (CNF1) to counteract epileptiform phenomena in inbred DBA/2J mice, an animal model displaying genetic background with an high susceptibility to induced- and spontaneous seizures. Via modulation of the Rho GTPases, CNF1 regulates actin dynamics with a consequent increase in spine density and length in pyramidal neurons of rat visual cortex, and influences the mitochondrial homeostasis with remarkable changes in the mitochondrial network architecture. In addition, CNF1 improves cognitive performances and increases ATP brain content in mouse models of Rett syndrome and Alzheimer's disease. The results herein reported show that a single dose of CNF1 induces a remarkable amelioration of the seizure phenotype, with a significant augmentation in neuroplasticity markers and in cortex mitochondrial ATP content. This latter effect is accompanied by a decrease in the expression of mitochondrial fission proteins, suggesting a role of mitochondrial dynamics in the CNF1-induced beneficial effects on this epileptiform phenotype. Our results strongly support the crucial role of brain energy homeostasis in the pathogenesis of certain neurological diseases, and suggest that CNF1 could represent a putative new therapeutic tool for epilepsy.

## Introduction

Epilepsy is caused by a variety of factors and is characterized by the abnormal firing of neurons, and by frequent seizures that bring about progressive damage to the brain. The abnormal synchronized discharge of a large number of neurons leads to a great consumption of bio-energy in the brain. [1,2]. On the other hand, mutations affecting genes encoding for proteins that maintain energy homeostasis within the cell, often result in an epileptiform phenotype [3,4]. This implies that energy failure can strongly affect neuronal excitability and synaptic transmission, thus contributing to epileptogenesis [5,6].

Furthermore, one of most deleterious seizure effect is the loss of dendritic spines. This latter feature was evidenced in both pathological specimens from animal seizure models [7,8], and human epilepsy patients [9,10], and was connected to depolymerization of actin [11]. Actin dynamic is a key regulator of the shape and morphological plasticity of axons, dendrites, and dendritic spines, and is controlled by the Rho family of small GTPases [12,13]. Interestingly, the Rho GTPases also orchestrate the close relationship between the actin cytoskeleton and mitochondrial shape and function [14].

In this context, we propose, as a therapeutic tool against seizures, a bacterial protein toxin from *Escherichia coli*, named cytotoxic necrotizing factor 1 (CNF1) that is able to 'modulate' the Rho GTPases’ activation/degradation process [15,16]. Recently, we have demonstrated that CNF1, boosts *in vitro* the mitochondrial ATP production and promotes mitochondria elongation by phosphorylating Drp1, a protein member of the dynamin family of large GTPases that controls mitochondrial fission [17]. Interestingly, the recruitment of Drp1 to mitochondria is facilitated by the actin cytoskeleton activity [18,19]. All these results are in line with what we have demonstrated using CNF1 *in vivo*. In fact, a single intacerebroventricular (icv) injection of the purified bacterial toxin can increase spine density and length in pyramidal neurons [20], lower the levels of neuroinflammation markers, and improve cognitive performances in Rett syndrome (RTT) [21] and Alzheimer’s disease (AD) [22] mouse models. In particular, in the RTT pathological model, we observed a rescue of brain mitochondrial electron transport chain activity [23] and an augmented expression of proteins involved in ATP regeneration [21]. In the AD mouse model, CNF1 promotes a systemic energy homeostasis rescue, with an increase of hippocampal and cortex tissue ATP content [22]. All these CNF1-induced effects are long lasting.

Possibly, the toxin can act by engaging pathways that control the actin cytoskeleton organization, thus increasing in neuroplasticity, and mitochondrial activity as well. Therefore, CNF1 may represent a therapeutic tool also for counteracting epilepsy or to preserve the brain from seizure-induced damage.

To evaluate the CNF1 ability to counteract seizures generation, we have studied CNF1 effects in an inbred strain of mice, the DBA/2J (D2). D2 is a multipurpose neurological disease model because of its susceptibility to disorders that may involve neuronal cell damage including glaucoma [24] and hearing loss [25]. In particular, D2 genetic background is also of particular interest because of the high susceptibility to induced [26–28] or spontaneous seizures compared to other inbred strains [29–33]. Our hypothesis is that the ability of CNF1 to enhance mitochondrial ATP cortical level by modulation of mitochondrial dynamics [17], may favour a cell energy restoration and may re-estabilish the correct neuronal function in cortices of this mouse seizure model, similarly to what occurs in pathological models with a cognitive impairment [21–23].

## Materials and Methods

### CNF1 preparation and treatments

CNF1 was obtained from the 392 ISS strain (provided by V. Falbo, Rome, Italy) and purified essentially as previously described [34] with a few modifications in the procedure. For all experiments, a concentration of 10^−10^ M CNF1 was used. The dose was selected following indications from previous papers [21–23].

### Ethical Guidelines

All procedures were carried out in accordance with the guidelines of the Council of European Communities and the approval of Bioethical Committee of the Italian National Institute of Health. All mice were housed in a central facility and maintained under controlled conditions of normal humidity and temperature, with standard alternating 12-h periods of light and darkness. Animals had free access to water and food. Mucedola S.r.l. (Settimo Milanese, Italy) supplied the diet, which contained 3.95 kcal/g equivalent to assimilable 2.7 kcal/g.

### Animal surgery

**Fig 1:**
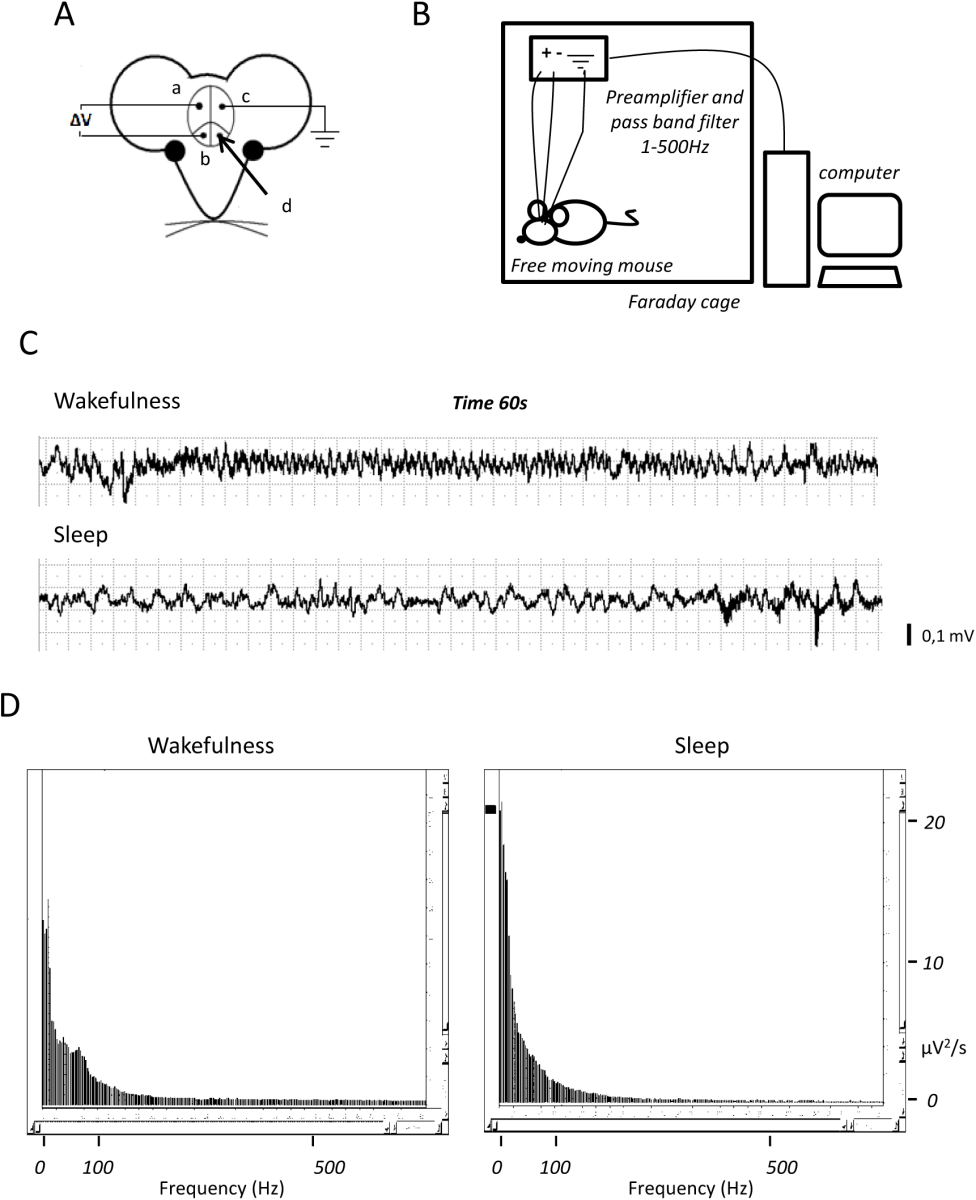
Cortical EEG and spectrogram analysis procedures. (A) Chronic cortical stainless steel electrodes (a) between right sensorimotor and visual cortex, (b) on the right frontal area, and (c) a ground electrode on the left sensorimotor area, (d) hole for icv injection of CNF1. (B) Digital amplifier-recording system. (C) EEG example tracks registered in awake (upper) and in sleep state. (D) Spectral analysis of 60 seconds of EEG traks from mice in wakefulness (left panel) and sleep state (right panel).

Experimental animals were male inbred DBA/2J mice, aged 20–25 weeks, purchased from Charles River Italia (Calco-Lecco, Italy). At least 8 days after arrival, mice received general (xylazine-ketamine) and local (lidocaine) anaesthesia, and were placed in a stereotactic apparatus. As schematized in Fig 1A, mice were implanted with chronic cortical stainless steel electrodes on the left sensorimotor area (c), between the right sensorimotor and visual cortex (a) and on the right frontal area (b), according to previously described techniques [35]. During the surgery approach, a hole was drilled in the left cortex area (Fig 1A and 1D), and a microsyringe connected to a micropump set at a flow-rate of 0.5 ml/min, was inserted through the brain cortex down to the left cerebral ventricle (Final injection coordinates: AP 0.1 mm, L 0.9 ¸V 2.1 mm from bregma). Through the syringe, 2 μl of sterile saline solution were injected into ten animals (control mice, saline). In random sequence, in ten additional animals, 2 μl of a 10^−10^ M CNF1 solution (treated mice, CNF1) were injected. Two minutes after the end of injections, the needle was removed and the surgical wound was sutured. CNF1 administration followed indications from previous papers [21–23].

### Recording of cerebral electrical activity

At least ten days after surgery, mice underwent recording of the cerebral electrical activity in steady-state conditions (EEG), in a sound-light- and electrically-shielded room, always in the same hours of the day (10:00–13:00), according to previously published procedures, with some modifications [36]. EEGs were recorded in periodograms of 600s and two physiological states were considered and acknowledged: wakefulness and slow sleep states. Briefly, mice electrodes were connected to a digital amplifier-recording system, set up in our laboratories with the technical assistance of Analysa (Cuneo, Italy) (Fig 1B). Signals from the two derivations of the right hemisphere were amplified (1000x), band-pass filtered (1 to 500 Hz) sampled at 2.5 kHz and recorded on disk in contiguous periodograms of 600s. The procedure was repeated 4 to 8 times, allowing a pause of 5 min between periodograms in three different sections (10, 15 and 20 days after surgery), alternating mice from the two groups, in order to have EEGs in the same hours of the day. In each mouse 8 to 16 periodograms were recorded, *i*.*e*., up a total of 80–160 minutes.

### EEG elaboration

EEG Tracks were examined by two different investigators in single blind condition considering frequency and amplitude patterns for visual evaluation. Then, EEG tracks were spectrally analyzed using a fast Fourier transform (FFT) algorithm to obtain their power spectral density function (PSD). Spectral density was computed on contiguous periods of 2024 points, with 90% overlapping between successive periods, and averaged [31,37].

To confirm visual identification of the different physiological states, EEGs showing wakefulness pattern were put in contiguous periodograms of 600s, which were then elaborated; tracks showing EEG sleep patterns were put in separate contiguous periodograms as well. Fig 1C shows examples of 60 seconds from EEGs registered in awake and in sleep state, respectively. Thus, EEG tracks also underwent spectral analysis, where sleep track presents higher spectral power in lower frequencies whereas wakefulness period showed typical theta frequencies peak pattern [31]. (Fig 1D).

**Fig 2:**
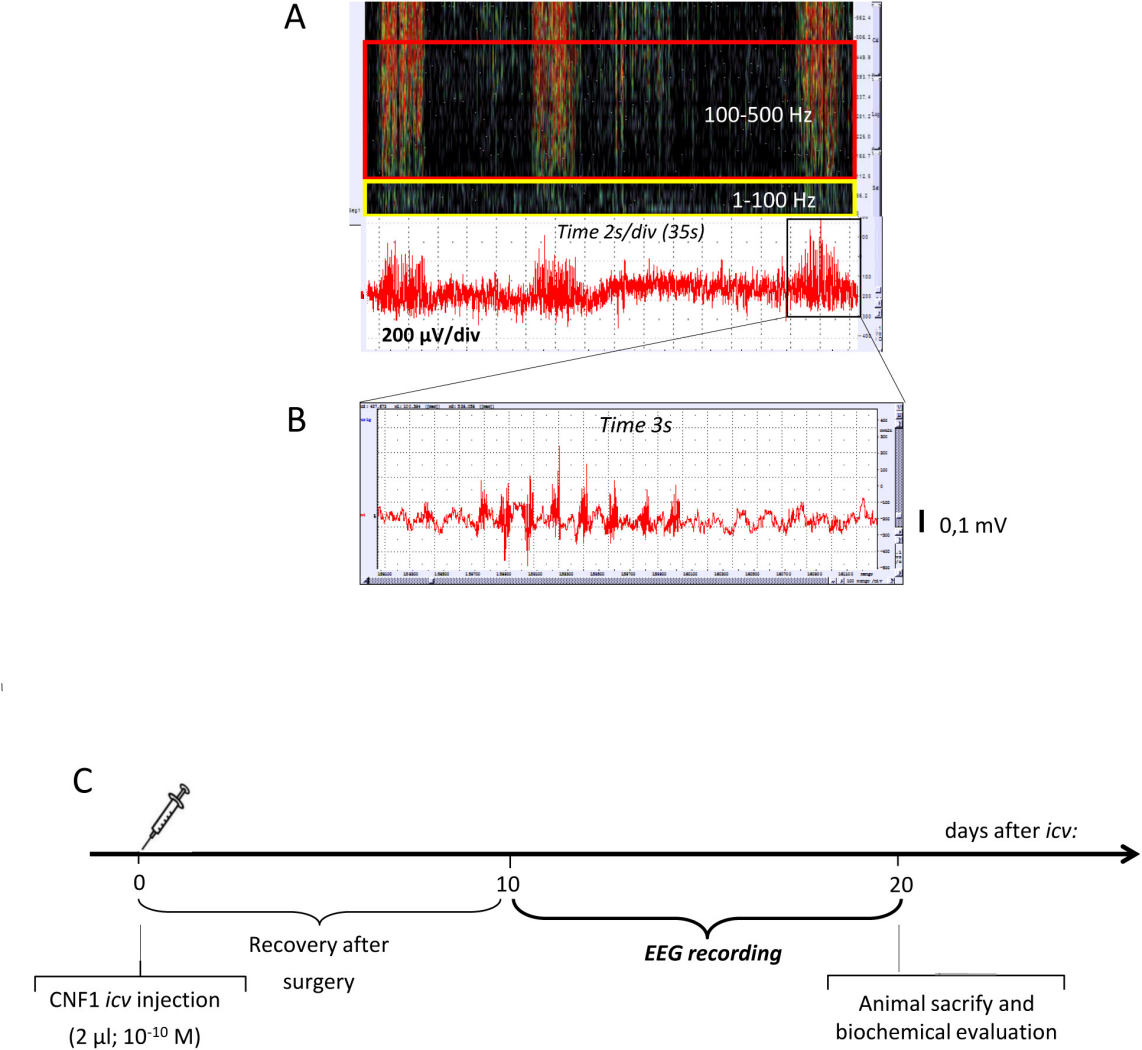
Frequency-and-time domain analysis procedures and experimental protocol. (A) Example of EEG (lower part of figure) and the calculated spectrogram (higher part of figure). Please note high frequency power values in the spectrogram, corresponding to series of polyspikes in the EEG. Spectral plot was set at 1–100 Hz (yellow square) and at 100–500 Hz (red square). (B) Enlarged record (total recording time 3s) of a seizure episode recorded in Fig 2A, showing a short episode of spontaneous polyspike-and-wave discharge, which is one of the typical seizure pattern recorded in the adult DBA/2J mouse. (C) Experimental protocol and time course.

EEG spectrograms were also elaborated according to a protocol of time and frequency-domain analysis, and parameters set up and published from our laboratories [38,39]. Briefly, spectrograms were recalled on the display, and elaborated according to the Soundscope protocol, in blocks of 600s. The protocol shows the powerspectral analysis of the entire 600s block through parallel vertical lines, which represent the power of spectral bands in pseudocolor, where the higher frequencies of the bands are expressed in dark colors (lower power bands) or in brilliant colors (yellow-red) that correspond to higher spectral power, from 1 to 500Hz. Analysis parameters are set according to the specific target for each investigation, including the physiologic states of the animal. In the present investigation, the spectral plot was set at 1 to 100 Hz and at 100 to 500 Hz, in lines computed on blocks of 256 consecutive points of the original tracing. The latter frequency band (100–500 Hz) is here defined as HFOs, i.e., high frequency oscillations. EEG low frequencies give a black display appearance, while high frequencies are colored, up to a maximal expression over the whole display covered with red-yellow lines. Data are gathered through imaging analysis (NIH imaging, http://rsb.info.nih.gov/nihimage/); full spectrogram corresponding to 600sec, is filled with high frequencies showing a lot of color. Wakefulness or sleep EEG tracks were gathered from the sleep-wakefulness continuum, were put together in sequence and averaged, with a mean duration of 20 min per mouse, 10 mice per group. Fig 2A shows a sample of 35s of EEG of saline-treated mice, with 3 episodes of polyspikes (Fig 2B shows a sample of 3s from same 600s periodogram to highlight epileptiform phenomena) evidenced in the lower part of the figure, while in the upper part of the figure the time-frequency domain analysis shows three series of bright color lines, corresponding to increased power of higher frequency bands (limited by the red rectangle superimposed). About ten days after electrophysiological registration, the animals were sacrificed after general anesthesia, with a mixture of ketamine and xylazine (85 and 15 mg/ml, respectively; 0.15 ml, sub cutaneous), and cortices were frozen using liquid nitrogen and stored at -80°C (Fig 2C).

### Western blot analysis

Frozen tissues were homogenized in 50 mM Tris (pH 7.4), 1 mM EDTA (pH 8.0), 0.5% Nonidet P-40, 150 mM NaCl, 10% glycerol, 10 mg/ml aprotinin, 10 mg/ml leupeptin, 1mM PMSF, 1 mM Na_2_VO_4_. Twenty-five micrograms of total protein extracts were resolved by SDS-polyacrylamide gel electrophoresis (PAGE) and electrically transferred onto polyvinylidene difluoride membranes (Bio-Rad). Membranes were blocked with Tris-buffered saline-Tween 20 (TBS-T) (20 mM Tris-HCl, pH 7.4, 150 mM NaCl, and 0.02% Tween 20) containing 5% skimmed milk (Bio-Rad, Hercules, CA) for 30 min at room temperature, and then they were incubated overnight at 4°C with primary antibodies diluted in TBS-T containing 2% milk. The following primary antibodies were used: rabbit polyclonal anti-spinophilin (Upstate, Lake Placid, NY; 1:1000), mouse monoclonal anti-PSD-95 (BD Biosciences, San Jose, CA; 1:250), mouse monoclonal anti-OPA1 (BD Biosciences, San Jose, CA; 1:1000), rabbit polyclonal anti-Mitofusin-2 (Cell Signaling Technology, Boston, MA; 1:1000), rabbit polyclonal anti-hFis1 (Enzo Lifesciences, Plymouth meeting, PA; 1:1000), mouse monoclonal anti-Drp1 (BD Biosciences, San Jose, CA; 1:1000), rabbit polyclonal anti-phospho-Drp1 (Cell Signaling Technology, Boston, MA; 1:1000), and anti-alpha-tubulin (Sigma-Aldrich, St Louis, MO; 1:10000). After extensive washing, immune complexes were detected with horseradish peroxidase-conjugated species-specific secondary antibodies (Jackson Laboratory, Bar Harbor, ME) followed by enhanced chemiluminescence reaction (Millipore Corporation, Billerica, MA). Proteins detected by immunoblotting were quantified by densitometry (ChemiDoc imaging system, BioRad).

### Cytochrome *c* oxidase assay

Frozen brain tissue were homogenized in cold mitochondrial buffer (210 mM mannitol, 70 mM sucrose, 1MM EDTA, 10 mM Hepes KOH pH 7.5) and proteases inhibitor cocktail (Sigma) in a Potter homogenizer with a Teflon pestle, as described [40]. The homogenate was centrifuged at 600 *g* for 10 min at 4°C. The supernatant was then transferred to glass centrifuge tubes and centrifuged at 7000g for 10 min at 4°C. After washing of the pellet in the same buffer, the pellet containing mitochondria was resuspended in the mitochondrial buffer and protein content determined by Bradford assay. Fifty micrograms of proteins were used to assess cytochrome *c* oxidase (Cytox) activity through spectrophotometric assay, following the oxidation of reduced cytochrome *c* (from horse heart; Sigma) (0.02 mmol/L) at 550 nm, as previously described [41]. A Beckman DU650 spectrophotometer (Beckman-Coulter, Fullerton, CA, USA) was used. Activity was expressed as nmol/min mg protein.

### Measurement of ATP content

Measurement of tissue ATP was performed using the ATP lite Assay (Perkin Elmer-Cetus, Norwalk, CT, USA). In brief, frozen tissues were homogenized in 50 μl of lysis buffer and mixed for 10 min. Forty microlitres of substrate solution (Luciferase/Luciferin) was added to each sample. The luminescence was measured using a luminescence plate reader (Victor3-V, PerkinElmer Life Sciences). The ATP concentration was normalized to total tissue protein concentration estimated by Bradford protein assay (Bio-Rad, Hercules, CA).

### Statistical analysis

Data was presented as Mean ± SEM. Independent samples t-test were used. Correlation was considered as significant when the *p* value was <0.05.

## Results

### CNF1 counteracts cortical spontaneous spike and wave discharges and high-frequency oscillations in aged DBA/2J mice

**Fig 3:**
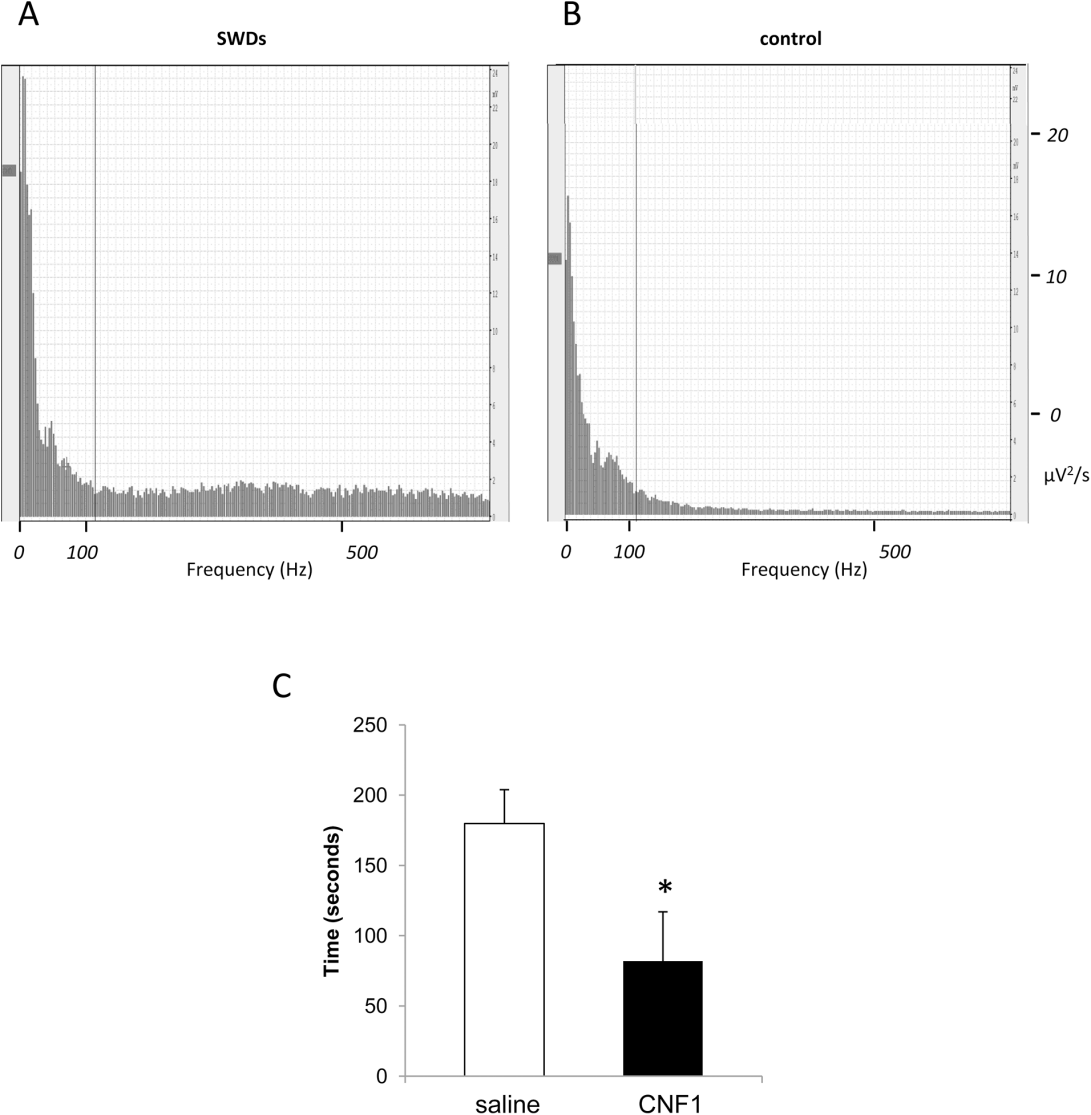
Spectral analysis of SWD and differences in SWDs between saline- and CNF1-treated mice. (A) Example of a 5 seconds SWD episode track evaluated through spectral analysis. Please note the differences in frequency wave between an SWD EEG track with respect to a control track. (B) Differences in total sum in time (seconds) of SWDs between saline (n = 6) and CNF1-treated mice (n = 6), * for p<0.05. Graphs report means ± SEM.

Spike and wave discharges **(**SWDs), which are expressed in aged D2 [31,32], where identified and expressed in total seconds over the total EEG recording time (1200 s), in six mice (25 week-old mouse) from both experimental groups in wakefulness condition. After the CNF1 icv injection, D2 showed a significant (p = 0.044) decrease of SWDs in seconds (179.8 ± 24.08) with respect to the saline-treated group (82.00 ± 34.96) (Fig 3C).

Fig 3A shows a spectral plot computed on 5 s of SWDs, and Fig 3B is representative of 5 s of a control EEG track. Please note that higher frequencies (100 to 500 Hz) are prevalent in the SWDs spectrum. This result appears in line with findings that show a direct link between SWDs, from absence seizure patients and animal models, and high frequency oscillations (HFOs) [42–44].

Thus, to evaluate the CNF1 effects against seizures generation, we analyzed the HFOs that have been implicated in many forms of seizures (spikes, poly-spikes, spike-and-slow wave sequences) [45,46] and that are expressed in aged D2 mice [38]. In fact, HFOs are preferentially localized in the brain region generating spontaneous seizures, and have been considered a potential biomarker of epileptogenic brain [45]. In the epileptic hippocampus, the activity from 100 or 200Hz to 500 Hz (or 250 Hz to 600 Hz) frequency band (fast ripples) is always considered epileptic and has been used as reliable marker of epileptic tissue in all hippocampal subregions. The relative rate of ripple frequency oscillations is a potential biomarker for epileptic neocortex and, in fact, HFOs seem to play an important role in seizure genesis and can be a useful clinical marker for disease diagnosis [47,48].

**Fig 4:**
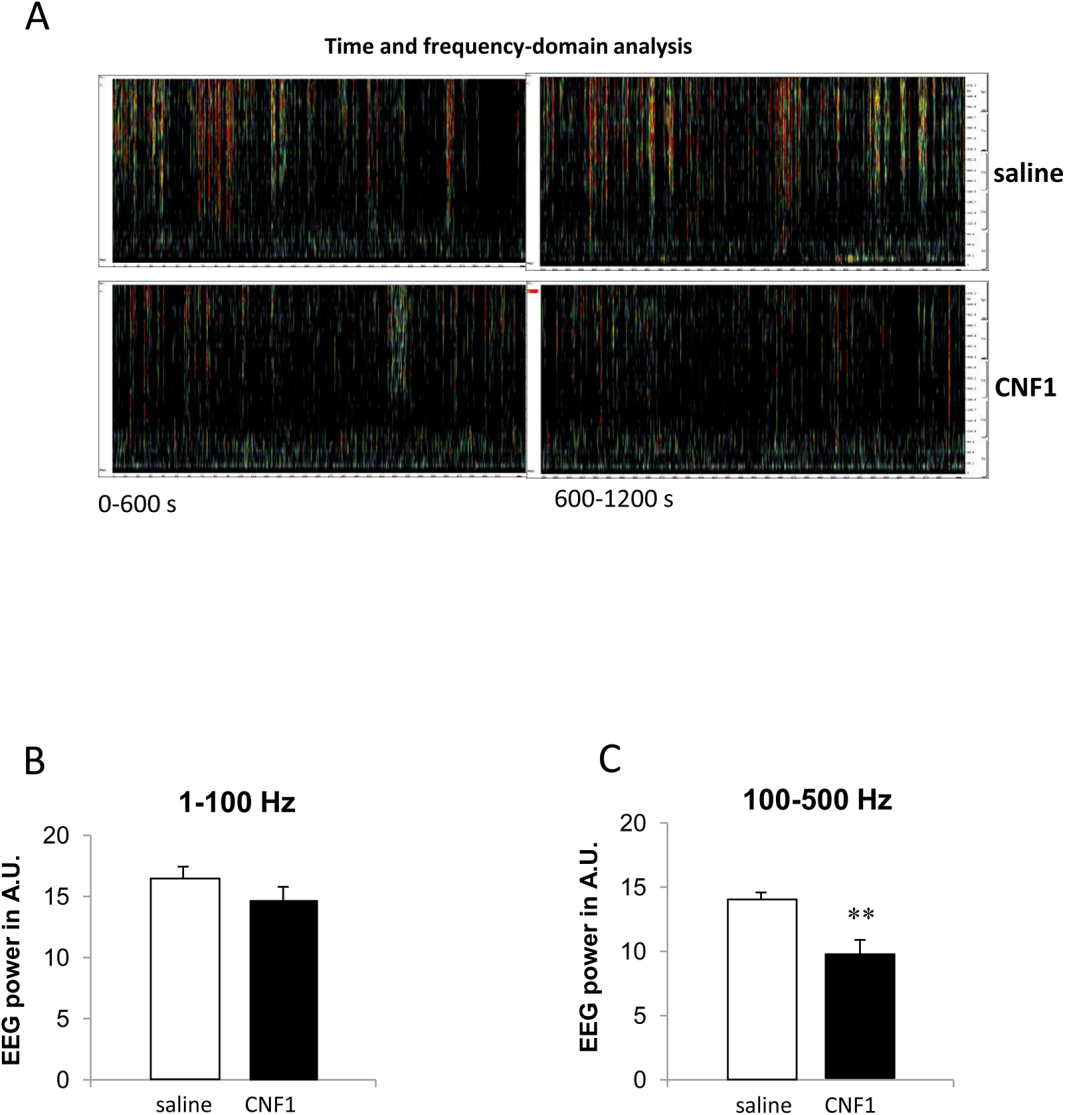
Differences in HFOs between saline and CNF1-treated mice. (A) Two calculated spectrograms from periograms of 600s recorded in a representative pathological mouse (25 week-old mouse) treated with saline or with CNF1 (respectively upper and lower part of the figure). Please note the presence of HFO phenomena in the 25 week-old control mouse, phenomena that are greatly decreased in the treated mouse. (B and C) Data from imaging analysis of frequency-and-time domain in the somatosensory cortex of control vs CNF1-treated mice. (B) Band 1–100 Hz, saline (n = 10) vs CNF1 (n = 10). (C) Band 100–500 Hz saline (n = 10) vs CNF1 (n = 10), ** for p<0.01. Graphs report means ± SEM.

Since there are differences in HFO rates in animal models and patients with seizures during the sleep/wakefulness cycle [29,49,50], cortical EEGs have been recorded in both physiological phases. We examined electroencephalogram (EEG) and the evaluation in frequency and time domain in somatosensitive cortex of 25 week-old D2 mice. Fig 4A shows samples of spectrograms calculated from two 600s periodograms recorded from a representative saline-treated pathological mouse model with a high presence of HFOs, versus samples of spectrograms calculated from two 600s periodograms recorded in wakefulness condition in a representative CNF1-treated mouse. Fig 4B shows the data from spectrograms in pathological control (16.3±1.1) and CNF1-treated pathological mice (14.6±1.2) in wakefulness condition, in the frequency band from 1 to 100 Hz, (p = 0.18, not significant), while Fig 4C shows the differences between data from spectrograms in wakefulness condition in control (13.8±0.7) and CNF1-treated mice (9.741±1.153) in the frequency band from 100 to 500 Hz. Statistical analysis showed a significant decrease of high frequency episodes from 100 to 500 Hz in wakefulness condition (p = 0.003).

### CNF1 increases neuroplasticity markers in aged DBA/2J mouse cortex

**Fig 5:**
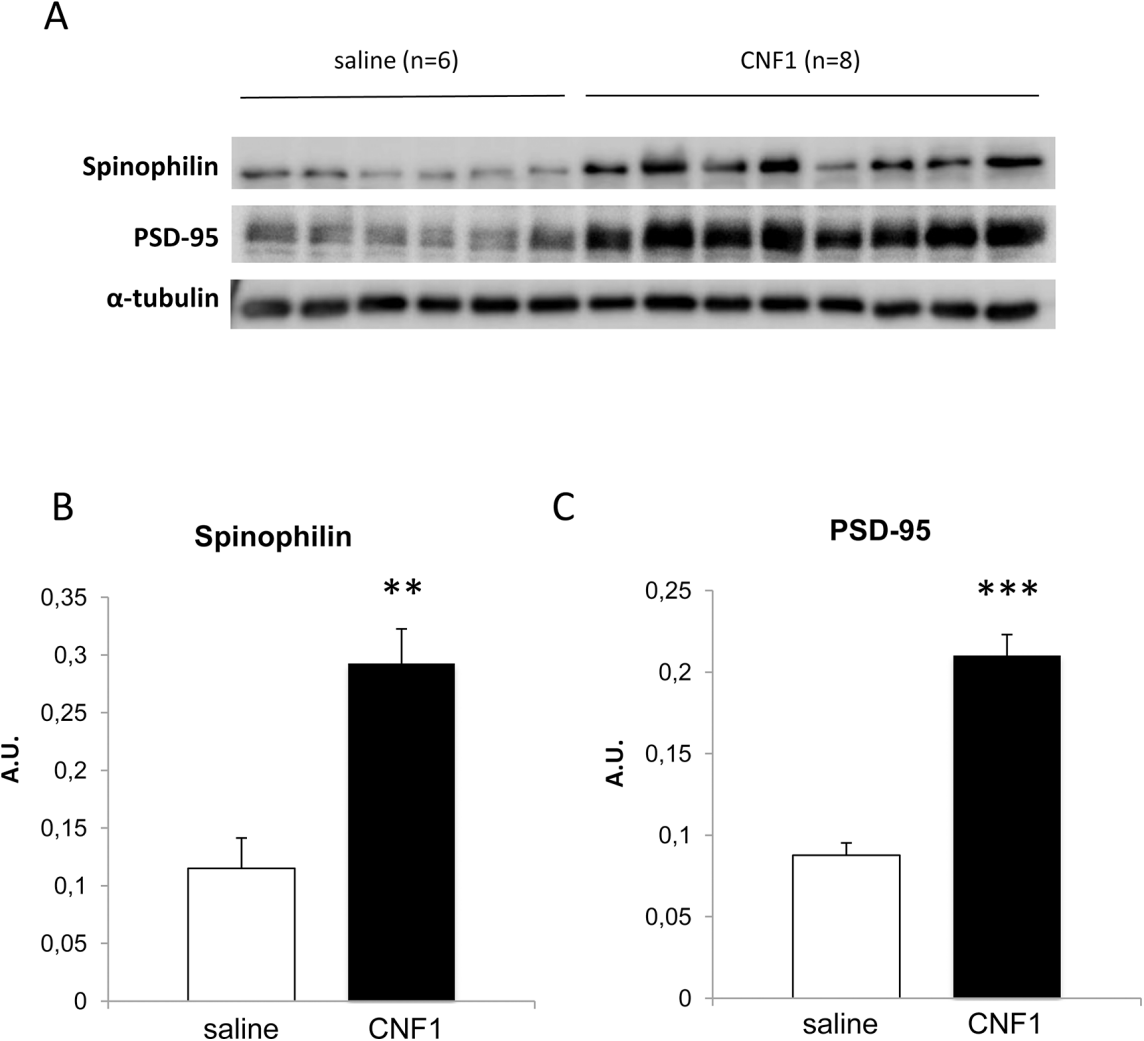
CNF1 effect on the expression of neuroplasticity markers in cortices of 25 week-old mice. (A) Representative Western Blot of spinophilin and PSD-95 protein expressions in cortex tissue (saline, n = 6; CNF1, n = 8). The amounts of the above proteins are normalized as a function of α-tubulin (histograms, B and C). CNF1 treatment induces a significant increase of both spinophilin (B) and PSD-95 (C) protein content. ** for p < 0.01 and *** for p < 0.001.

To investigate whether the CNF1 ability to trigger brain functional plasticity is involved in the observed decrease of seizures, we measured the spinophilin and postsynaptic density-95 (PSD-95) levels in cortices of the saline- and CNF1-treated mice (Fig 3). PSD-95 is a well known marker of synaptic size [51] and its expression decreases after seizures [52]. Spinophilin interacts with several proteins that are highly enriched in spines [53,54]. One of them, actin, is important for the formation, maintenance, and morphology of spines [55]. *In vitro*, spinophilin bundles actin filaments [53], suggesting its possible role as an organizer of the actin-based cytoskeleton in dendritic spines [55,56]. Investigating about the CNF1 effect on synaptic plasticity in this pathological model, we found that both spinophilin and PSD-95 proteins were significantly (p = 0.0011 and p<0.0001, respectively) overexpressed in CNF1-treated mice (0.29±0.,03 and 0.21±0.013, respectively) with respect to control mice (0.11±0.03 and 0.09±0.008, respectively) (Fig 5), suggesting that the toxin can influence dendritic spines functionality.

### CNF1 inhibits mitochondrial fission and increases mitochondrial ATP level in the cortex of aged DBA/2J mice

To confirm the CNF1 ability to trigger brain energy production, due to a modulation of mitochondrial dynamics, we analyzed the expression of proteins involved in mitochondrial fusion (Mfn2 and OPA1) and fission (hFis1 and Drp1) processes, and ATP content in all mice cortices. The amount of Drp1 phosphorylated at Ser637, a post-translational modified protein that stimulates mitochondrial elongation and unopposed fusion [57,58] was also determined.

**Fig 6:**
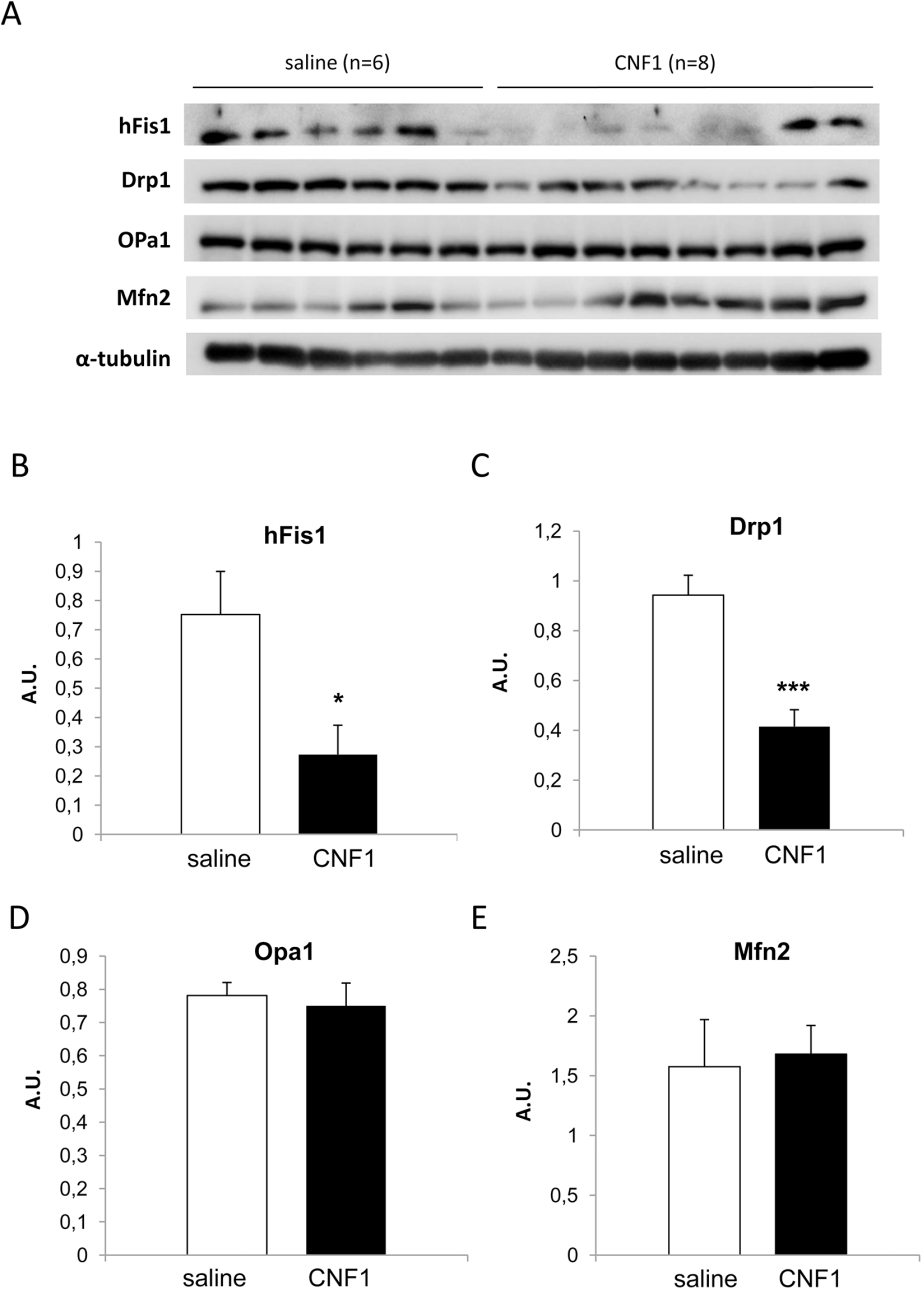
CNF1 effect on proteins involved in mitochondrial dynamics in cortex tissues from pathological mice. (A) Representative Western Blot of fission and fusion proteins in cortex tissues from 25 week-old mice (saline, n = 6; CNF1, n = 8). The amounts of the above proteins are normalized as a function of α-tubulin. CNF1 treatment induces a significant decrease of both fission proteins hFis1 (B) and Drp1 (C), while not modifying fusion markers expression Opa1 (D) and Mfn2 (E). * for p < 0.05 and *** for p < 0.001.

Results reported in Fig 6 clearly show that the expression of fission-related proteins in pathological mice brain cortices [hFis1(0.75±0.15) and Drp1 (0.94±0.08)], is significantly reduced following *icv* injection of CNF1 (0.27±0.1 and 0.41±0.07, respectively; p = 0.02 for hFis1 and p = 0.0003 for Drp1). In contrast, fusion markers (OPA1 and Mfn2) were not modified (p = 0.73 for Opa1 and p = 0.82 for Mfn2) by toxin challenge.

**Fig 7:**
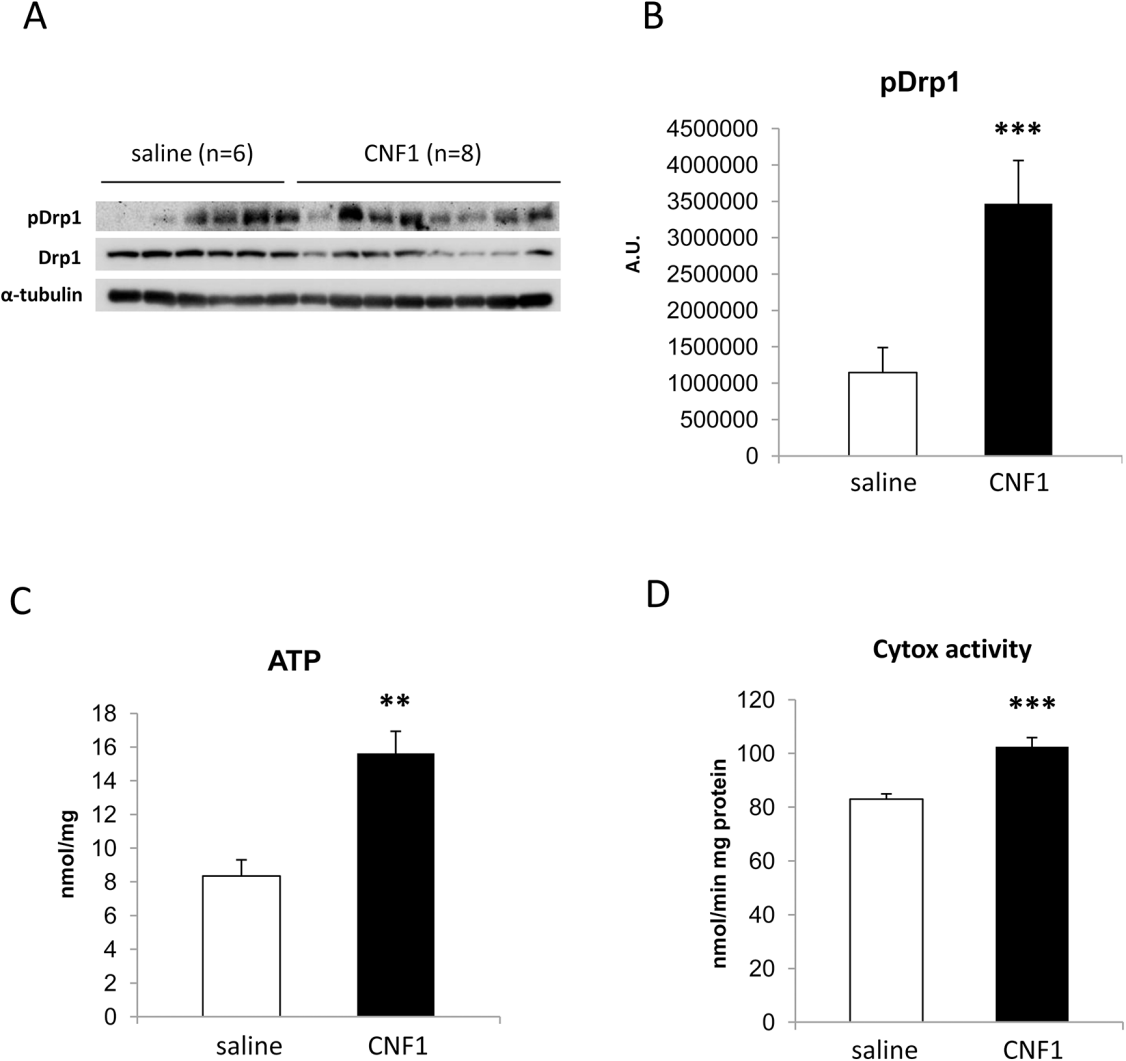
CNF1 effect on Drp1 phosphorylation and energetic content in pathological mouse model (25 week-old) cortices. (A) Representative Western Blot of Drp1 phosphorylated in Ser637 (pDrp1) in cortex tissue (saline, n = 6; CNF1, n = 8). The amount of pDrp1 is normalized as a function of Drp1, in turn normalized on α-tubulin (histogram, B). Note that CNF1 induces a significant augmentation of pDrp1 expression. (C) Differences in ATP cortex content, measured by a luminometric assay, between controls (n = 10) and CNF1-treated (n = 10) mice. (D) Differences in cytochrome *c* oxidase activity measured by spectrophotometric assay, between controls (n = 6) and CNF1-treated (n = 6) mice. The graphs report means ± SEM. * for p < 0.05, ** for p < 0.01 and *** for p < 0.001.

Moreover, as demonstrated in our previous work *in vitro* [17], we found that during CNF1 treatment the amount of Ser637-phosphorylated Drp1 was significantly increased (p = 0,0096) in cortices of CNF1-treated mice (3467000 ± 595200) with respect to control mice (1146000 ± 343700) (Fig 7A and 7B). All in all the above results suggest that CNF1 can inhibit mitochondrial fission by both modifying specific protein expression and acting on their post-translational processing.

Analyzing the ATP levels in mice cortices (Fig 7C), we found a significative (p = 0.0012) increase in ATP content in CNF1-treated mice (15.6±1.3) *vs* control (8.3±0.96) samples. This finding corroborates the ability of CNF1 to favor the cell energy restore in mice and, more importantly, is the first demonstration that such a toxin property is not linked to cognitive defects but can re-establish neuronal functions also in a mouse model without cognitive impairment. The analysis of cytochrome *c* oxidase (Cytox) activity following CNF1 treatment, confirms that the cortical tissue energy increase derives from an augmentation of the mitochondrial electron transport chain activity. In fact, CNF1 increased significantly (p = 0,0002) cortical Cytox activity of D2 (102.4 ± 3.464) *vs* control group (82.95 ± 1.134) (Fig 7D).

## Discussion

The present study was designed to explore the effects of the Rho GTPases’ modulator CNF1 on deputy biomarkers for seizures in the the neocortex. Particularly, we investigated the CNF1 effect on energy homeostasis and brain plasticity in the inbred strain of mice DBA/2J, a multipurpose neurological disease model that presents high susceptibility to induced [26–28] or spontaneous seizures [29–33], and the presence of spontaneous fast ripples [38]. We herein report that a single *icv* injection of CNF1 is able to raise a consistent and enduring antagonism to the spontaneous cortical EEG SWDs and HFOs (100–500 Hz range) during the physiological state of wakefulness in six-month-old DBA/2J mice. Such an effect is accompanied by an increase in PSD-95 and spinophilin expression, a decrease in the expression of mitochondrial fission proteins hFis1 and Drp1 as well as an increase in the mitochondrial ATP content and in the phosphorylated form of Drp1 (pSer637-Drp1) in brain cortex tissue.

Seizures are present in a number of CNS diseases and are frequently associated with high energy demand, requiring both rapid adaptation of altered oxidative energy metabolism and sufficient supply with oxygen and nutrients [59–62]. In the same way, synaptic plasticity homeostasis is involved in epileptogenesis [63] and in epileptic phenomena, such as SWDs [64,65]. In fact, gliotransmitters like ATP can modulate postsynaptic GABA receptors, which are involved in SWDs [65] and in epilepsy in general [66]. In the same way, data on spinophilin and PSD 95, which show an increment in brain plasticity, and are also probably strictly linked to an increase in energy production. As a matter of fact, mitochondria play important roles in controlling fundamental processes in neuroplasticity, including neural differentiation, neurite outgrowth, neurotransmitter release and dendritic remodeling, through energy generation (ATP and NAD+) and subcellular Ca^2+^ and redox homoeostasis regulation. Since ATP and NAD+ are a major source of energy, required for maintenance and restoration of ion gradients, mitochondria are essential components in synaptic transmission [67]. Thus, an increase in ATP production may trigger an increase in synaptic plasticity and a decrease in seizure generation.

On these bases, as previously demonstrated *in vitro* in epithelial cells and in brain of RTT models, also in an epilepsy mouse model it is reasonable to speculate a main activity of CNF1 on mitochondrial dynamics and ATP production. The increase in ATP production may down-regulate seizure generation with a decrease in HFO pattern and in seizure frequency. In addition, CNF1 may trigger synaptic plasticity, with an increase in “functional” dendritic spines. Of course, a decline in epileptic phenomena may also allow a lower consumption of ATP probably enhancing CNF1 therapeutic effectiveness. This hypothesis is in line with that formulated by other authors, who consider the increase in ATP production, through aerobic metabolism and/or anaerobic glycolysis, as a promising new neuroprotective strategy in epilepsy, with a direct link to seizure generation [68, 69].

## Conclusions

Mitochondrial dysfunctions are considered a potential cause of both epileptic seizures and therapy-resistant forms of severe epilepsy [70] since the impairment of mitochondrial function is present in the seizure focus of human and experimental epilepsy [71]. More recently, it has been reported that a broad variety of mutations of mitochondrial DNA, which lead to the inhibition of mitochondrial oxidative phosphorylation selectively in epileptogenic areas of the human brain, have been associated with epileptic phenotypes [4]. Hence, this work demonstrates that the increase in ATP content triggered by CNF1 occurs in a pathological mouse model that does not present cognitive defects [72]. This strongly supports our hypothesis that CNF1 can be regarded as a new potential bioenergetic approach for CNS diseases with energy deficits. These observations point at CNF1 as both a new neuroprotective strategy against seizures disorders and a novel potential treatment for therapy-resistant forms of epilepsy.
